# Controlled low central venous pressure in laparoscopic left hemihepatectomy for left hepatolithiasis with prior upper abdominal surgery: A retrospective cohort study

**DOI:** 10.1097/MD.0000000000043216

**Published:** 2025-07-11

**Authors:** Dingxiu He, Yuting Fan, Jinghang Liu, Huizhen Chen, Wei Hu, Weihua Pan, Longjian Ran, Lu Fang, Jian Huang

**Affiliations:** a Department of Anesthesia, The Second Hospital of Longyan, Longyan, Fujian, China; b Department of General Surgery, The Second Affiliated Hospital of Nanchang University, Nanchang University School of Medicine, Nanchang, Jiangxi, China; c Department of Hepatobiliary Surgery, Nanyang First People’s Hospital, Henan, China; d Department of Respiratory, Shanghang County Hospital, Shanghang, Fujian, China; e Department of Hepatobiliary Surgery, Xiaogan Central Hospital, Xiaogan, Hubei, China; f Department of Hepatobiliary Surgery, The Second Hospital of Longyan, Longyan, Fujian, China; g Department of Hepatobiliary Surgery, The Second Affiliated Hospital of Nanchang University, Nanchang, Jiangxi, China.

**Keywords:** controlled low central venous pressure, hepatectomy, hepatolithiasis, previous operative history

## Abstract

This study aimed to evaluate the safety and effectiveness of controlled low central venous pressure (CLCVP) during middle hepatic vein (MHV)-guided laparoscopic anatomical left hemihepatectomy for patients with intrahepatic bile duct stones and a prior history of upper abdominal surgery. A retrospective analysis was conducted on the clinical data of patients who had undergone MHV-guided laparoscopic anatomical left hemihepatectomy for hepatolithiasis with a prior history of upper abdominal surgery. The patients were treated at the Second Affiliated Hospital of Nanchang University and the Second Hospital of Longyan between January 2018 and January 2024. The patients were divided into CLCVP group (0 ≤ CVP ≤ 5 cmH_2_O) and normal CVP (NCLCVP) group (5 < CVP ≤ 10 cmH_2_O). The preoperative, intraoperative and postoperative clinical data of the 2 groups were analyzed and compared. This study included 100 patients, 45 in the CLCVP group and 55 in the NCLCVP group. The 2 groups showed no significant disparities in baseline data. Conversion to open surgery occurred in 7 (7%), while the remaining procedures were successfully performed laparoscopically. Compared with the NCLCVP group, the CLCVP group had shorter operation time (303.00 ± 40.15 minutes vs 328.18 ± 51.43 minutes, *P* = .007) and less blood loss (185.56 ± 71.21 mL vs 260.00 ± 122.63 mL, *P* < .001). There was no significant difference in other observation indexes between the 2 groups. When preoperative liver function is evaluated and patients’ perfusion parameters are closely monitored during the procedure, CLCVP can be safely and effectively applied in MHV-guided laparoscopic anatomical left hemihepatectomy for intrahepatic bile duct stones patients with previous upper abdominal surgery. This technique contributes to reduced blood loss and a shorter operative duration.

## 1. Introduction

The liver has a rich vascular network in physiology. If the operation is improper during hepatectomy, it is prone to intraoperative bleeding, which directly threatens the life and health of patients.^[1,2]^ The application of hepatic vascular inflow control and anesthesia techniques in hepatic parenchymal transection can increase the probability of safe and rapid resection, which has been widely accepted.^[3]^ During hepatectomy, the central venous pressure (CVP) is maintained at 5cm H_2_O, combined with Pringle maneuver, which can effectively reduce the amount of bleeding in patients undergoing hepatectomy.^[4,5]^

Intrahepatic bile duct stones usually require active intervention due to their high carcinogenicity (significantly increasing the risk of cholangiocarcinoma) and the tendency to cause serious complications such as recurrent cholangitis, liver abscess, biliary cirrhosis, and portal hypertension. Therefore, for patients who are diagnosed, it is strongly recommended to consider surgical treatment to remove lesions, relieve obstruction, prevent canceration, and deal with related complications. The diagnosis of intrahepatic bile duct stones should be combined with multi-dimensional evaluation, including clinical manifestations (manifestations including recurrent right upper quadrant pain, jaundice, and cholangitis), imaging examinations [abdominal ultrasound, computerized tomography, magnetic resonance imaging (MRI) and magnetic resonance cholangiopancreatography (MRCP)] and laboratory indicators (abnormal liver function, elevated infection markers).^[6]^ In cases of extensive left-sided intrahepatic bile duct stones, anatomical left hepatectomy is indicated to ensure complete resection of both the affected parenchyma and calculi.^[7]^

Research indicates that middle hepatic vein (MHV)-guided laparoscopic left hemihepatectomy represents a safe and efficacious approach for managing left-sided intrahepatic choledocholithiasis in patients with prior upper abdominal surgical history.^[8–10]^ A large number of studies have shown that controlled low central venous pressure (CLCVP) technology can effectively reduce the amount of bleeding in the liver parenchymal section by reducing the pressure in the hepatic sinus and hepatic vein, thereby reducing intraoperative and postoperative blood transfusion, shortening hospital stay, and reducing postoperative complications, and has no significant effect on liver and kidney function.^[11–13]^ Currently, there is limited literature on the application of CLCVP technique in laparoscopic hepatectomy for patients with a history of upper abdominal surgery. This retrospective study aimed to evaluate the safety, efficacy, and feasibility of CLCVP technique in MHV-guided laparoscopic anatomical left hemihepatectomy for patients with left-sided intrahepatic bile duct stones and a prior history of upper abdominal surgery.

## 2. Materials and methods

### 2.1. Patients and grouping

**Figure 1.**
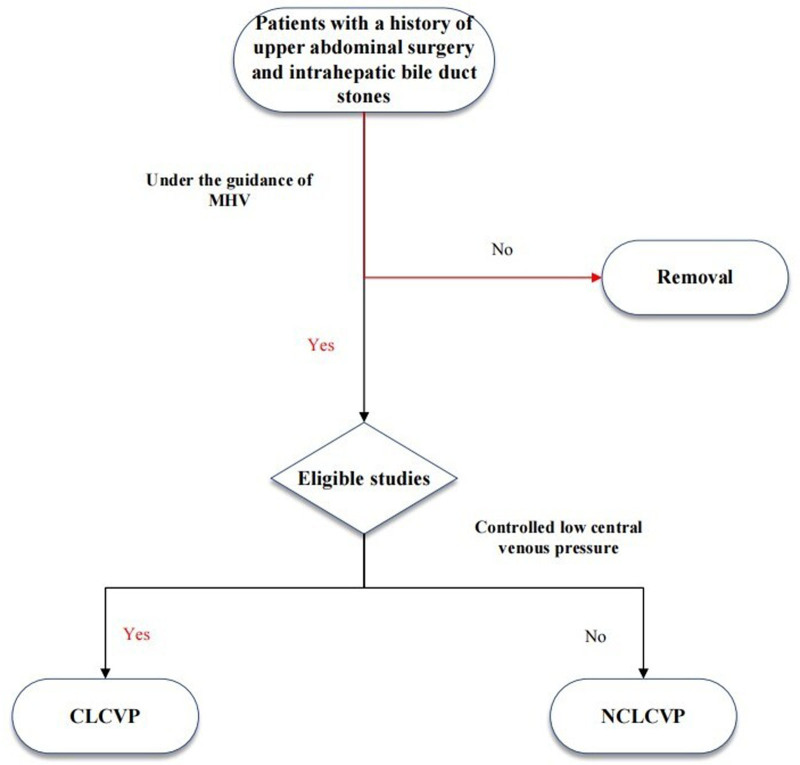
The flow chart of patient screening in this study.

A retrospective analysis of clinical data was conducted for 100 patients who underwent MHV-guided laparoscopic anatomical left hemihepatectomy for left-sided intrahepatic bile duct stones with a prior upper abdominal surgical history. The patients were treated at the Second Affiliated Hospital of Nanchang University and Longyan City Second Hospital between January 2018 and January 2024. For patients with extensive left hepatic calculi and a prior history of upper abdominal surgery, it is necessary to evaluate the anatomical variation of intrahepatic bile ducts through 3-dimensional reconstruction. In consideration of liver function reserve (assessed via Child–Pugh classification) and the distribution of stones, MHV-guided anatomical left hepatectomy is recommended to achieve complete lesion resection and minimize recurrence risk.^[14]^ At the same time, the patients were divided into CLCVP group (0 ≤ CVP ≤ 5 cmH_2_O) and normal CVP (NCLCVP) group (5 < CVP ≤ 10 cmH_2_O). The CVP management strategy was determined by anesthesiologists based on the patient’s preoperative cardiac function status (such as left ventricular ejection fraction, history of congestive heart failure) and surgical procedure. The specific criteria were as follows: patients without underlying heart disease were preferentially included in the CLCVP group, and CVP 0 to 5 cmH_2_O should be maintained; patients with hypertension or coronary heart disease were included in the NCLCVP group, and CVP 5 to 10 cmH_2_O was maintained to avoid insufficient perfusion of important organs. Preoperative diagnosis of patients was established based on clinical manifestations (including right upper quadrant pain, jaundice, and cholangitis), imaging studies (such as abdominal ultrasonography, computed tomography, and MRI), and laboratory test results. The main endpoints of this study: intraoperative blood loss, operation time; secondary endpoints: postoperative liver function [alanine aminotransferase (ALT), total bilirubin (TBIL)], renal function [glomerular filtration rate (GFR)], complication rate, stone recurrence rate. Inclusion criteria: extensive left liver stones; the existence of common bile duct stones; a past history of upper abdominal surgeries; Child-Pugh grade A or B liver function. Exclusion criteria: Child–Pugh C; severe dysfunction of cardiac, cerebral, renal, and other vital organs; previous biliary-enteric anastomosis; lack of complete case data. The flow chart of patient screening in this study is shown in Figure 1. This study was approved by the Ethics Committee of the Second Affiliated Hospital of Nanchang University and the Second Hospital of Longyan City. All subjects provided informed consent before participating in this study and used human tissues for research in accordance with established ethical guidelines.

## 3. Surgical approach

### 3.1. CLCVP group

Under general anesthesia with tracheal intubation, a 7 French (Fr) double-lumen central venous catheter was placed through the right internal jugular vein (preferred) or subclavian vein. The CVP is continuously monitored using an electronic pressure sensor (Datex Ohmeda monitor), with the fourth intercostal space of the midaxillary line as the zero point. The patient is placed in a supine position, and the head of the bed is adjusted to a horizontal position before reading the CVP value. Calibrate every 30 minutes to ensure data accuracy.

The patients were positioned in an anti-Trendelenburg orientation (15° head-up tilt), with pneumoperitoneum pressure maintained at 12 to 14 mm Hg (1 mm Hg = 0.133 kPa). The trocar placement followed a 5-hole configuration, with carbon dioxide pneumoperitoneum pressure maintained at 13 mm Hg (1 mm Hg = 0.133 kPa). The fluid infusion volume was controlled at 1 mL/(kg h) according to body weight 30 minutes before liver parenchymal transection. CLCVP method: nitroglycerin, norepinephrine, dopamine and other drugs were used to maintain CVP at 0 ≤ CVP ≤ 5 cmH_2_O, systolic blood pressure (SBP) > 90 mm Hg, mean arterial pressure (MAP) > 60 mm Hg during the same period. Anesthesia maintenance is performed using propofol (4–6 mg/kg/h) combined with remifentanil (0.1–0.2 μg/kg/min). The muscle relaxant uses cisatracurium and the dosage is adjusted based on Train of Four monitoring. Diuretics were used appropriately to ensure that the patient’s urine volume > 1 mL/ (kg h). After hepatectomy and complete hemostasis of the wound, the CVP was quickly restored and maintained above 6 cmH_2_O by 1:1 rehydration of crystal colloid.

The ligaments around the liver were dissociated by ultrasonic scalpel. The first hepatic portal vein was blocked by Pringle maneuver (Intermittent hepatic portal occlusion, normal liver 10 to 15 minutes each time, interval 5 to 10 minutes to restore blood flow, cumulative blocking time ≤ 90 minutes; liver cirrhosis, fatty liver 10 to 15 minutes each time, interval of 5 minutes to restore blood flow, cumulative blocking time ≤ 60 minutes; at the same time, according to liver function adjustment.) dissect the first porta hepatis, expose the left branch of the portal vein and the left hepatic artery and ligate them. The ischemic region was demarcated using an electric hook. Intraoperative ultrasonography was employed to localize the termination of MHV, followed by tracing its main trunk from the identified termination, which was then marked with an electric hook. Under the guidance of MHV, the liver parenchyma was transected in a caudo-cranial direction. Hemlock clips were applied to blood vessels or bile ducts with a diameter > 2 mm. Thereafter, upon exposing the root of the left hepatic vein, the vessel and residual liver parenchyma were resected using a linear cutter. During hepatectomy, the MHV was exposed, and CVP was maintained below 5 mm Hg. In case of venous wall bleeding from the MHV, ligation was performed with 5-0 Proline suture. The surgical site was inspected for bile leakage or active hemorrhage, followed by electrocoagulation or suture repair as needed.

The common bile duct was incised longitudinally or via the transected end of the left hepatic duct. Choledochoscopy was then performed to meticulously inspect the intrahepatic ducts (left and right hepatic ducts) and common bile duct. A metal stone retrieval basket was utilized to extract calculi. Following stone clearance, a T-tube was placed and secured based on intraoperative findings. Saline was injected through the T-tube using a syringe to assess for bile leakage at the common bile duct suture, the left hepatic duct stump, and hepatic segments. A liver specimen was retrieved via a 5-cm longitudinal subxiphoid incision. The abdominal drainage tube was positioned at the left hepatic section and Winslow foramen (the omental foramen, located behind the hepatoduodenal ligament, serving as a passage connecting the peritoneal cavity and the lesser omental sac. During surgery, it is used to place a drainage tube for monitoring exudate from the hepatic section^[15]^), The subxiphoid incision and trocar sites were then closed layer by layer.

Postoperatively, the patients were returned to the ward for continuous ECG monitoring for 48 hours, and the vital signs (heart rate, blood pressure, CVP, blood oxygen saturation) were monitored every 15 to 30 minutes. The liver function [ALT/aspartate aminotransferase (AST), TBIL], renal function (serum creatinine, blood urea nitrogen) and coagulation function were examined on the 1st, 3rd, and 5th day after operation. Observe the volume and color of abdominal drainage fluid, and keep the T tube unobstructed. Early activity (standing at the bedside within 24 hours after surgery), fluid diet on the first day after surgery. Be alert to bleeding, bile leakage, regular follow-up of liver function and abdominal imaging.

### 3.2. NCLCVP group

The preoperative preparation was the same as that of the CLCVP group. CVP was controlled within 5 < CVP ≤ 10 cmH_2_O during liver parenchymal transection. The remaining surgical procedures were identical to those in the CLCVP group.

### 3.3. Statistical analysis

We used IBM SPSS version 25.0 (SPSS Inc., Chicago) for the statistical analysis. All continuous variables were presented as means ± standard deviations and classification data as numbers and percentages. The Cochran–Mantel–Haenszel χ^2^ test was used to compare the demographic characteristics and clinical manifestations between the ICG group and the no-ICG group. *P* < .05 was considered to be statistically significant.

## 4. Results

This study included 100 patients, including 46 males (46 %) and 54 females (54 %), with an average age of 59.14 ± 9.35 years. The demographic characteristics of patients in the CLCVP group and the NCLCVP group were similar, as shown in Table 1. The prior surgical history of patients in both groups is specified in Table 2.

**Table 1:** Characteristics and clinical presentations of patients who underwent.

	CLCVP group (n = 45)	NCLCVP group (n = 55)	*P*-value
Sex, n (%)			
Male	25 (56%)	21 (38%)	.083
Female	20 (44%)	34 (62%)	
Age (yr)	59.98 ± 6.33	58.45 ± 11.26	.396
ASA classification, n (%)			
Ⅰ	7 (16%)	8 (15%)	.888
Ⅱ	30 (67%)	38 (69%)	.796
Ⅲ	8 (17%)	9 (16%)	.851
≥Ⅳ	0	0	
BMI (kg/m^2^)	22.39 ± 3.15	23.45 ± 3.41	.113
Child–Pugh class, n (%)			
A	39 (87%)	47 (85%)	.862
B	6 (13%)	8 (15%)	
C	0	0	
Diabetes, n (%)	7 (16%)	6 (11%)	.492
Hypertension, n (%)	8 (18%)	10 (18%)	.958
Hemoglobin (g/L)	123.89 ± 8.06	121.85 ± 5.38	.151
Prothrombin time (s)	12.54 ± 2.15	12.02 ± 0.79	.126
Alanine transaminase (U/L)	60.67 ± 15.12	54.65 ± 20.50	.095
Aspartate transaminase (U/L)	51.27 ± 16.94	58.21 ± 18.53	.055
Total bilirubin (μmol/L)	24.46 ± 11.85	34.75 ± 13.33	.909
Albumin (g/L)	39.78 ± 3.78	41.37 ± 4.86	.075
Scr (μmol/L)	63.18 ± 11.95	60.27 ± 10.72	.203
BUN (mmol/L)	5.22 ± 1.63	4.80 ± 1.80	.234
GFR [mL/(min·1.73 m^2^)]	96.09 ± 2.10	97.01 ± 4.42	.178
Combined with CBD stones, n (%)	35 (78%)	44 (80%)	.786

ASA = American Society of Anesthesiologists, BMI = body mass index, BUN = blood urea nitrogen, CBD = common bile duct, CLCVP = controlled low central venous pressure, GFR = glomerular filtration rate, NCLCVP = normal CVP, Scr = Serum creatinine.

**Table 2:** Types of previous upper abdominal surgery history.

	CLCVP group (n = 45)	NCLCVP group (n = 55)	*P*-value
More than 1 previous abdominal surgeries, n (%)			
No	35 (78%)	44 (80%)	.786
Yes	10 (22%)	11 (20%)	
Previous abdominal surgery, n (%)			
Partial hepatectomy	14 (31%)	16 (29%)	.826
LC	10 (22%)	9 (16%)	.458
LCBDE	15 (33%)	16 (29%)	.648
LCBDE + LC	12 (27%)	19 (42%)	.397
Gastrointestinal surgery	5 (11%)	7 (13%)	.805
Laparoscopic distal pancreatectomy	1 (2%)	2 (4%)	.680
Laparoscopic splenectomy	1 (2%)	4 (7%)	.249

CLCVP = controlled low central venous pressure, LC = laparoscopic cholecystectomy, LCBDE = laparoscopic common bile duct exploration, NCLCVP = normal CVP.

Intraoperative data are summarized in Table 3. Seven patients required conversion to open surgery due to severe abdominal adhesions or uncontrollable hemorrhage. Specifically, 3 patients in the CLCVP group had severe adhesions, while the NCLCVP group included 1 patient with adhesions and 3 with uncontrolled bleeding. The remaining patients underwent successful laparoscopic procedures. Compared with the NCLCVP group, the CLCVP group demonstrated significantly shorter operation time [303.00 ± 40.15 minutes (95% CI: 290.92–315.08) vs 328.18 ± 51.43 minutes (95% CI: 314.43–341.93), *P* = .007] and less intraoperative blood loss [185.56 ± 71.21 mL (95% CI: 164.70–206.42) vs 260.00 ± 122.63 mL (95% CI: 226.48–293.52), *P* < .001]. There was no significant difference in lactic acid between the 2 groups at the beginning of the operation. This difference began to appear when the hepatic portal was blocked, and the difference was the largest after hepatectomy. 79 (79.0%) had concomitant common bile duct stones. Of these, 25 (31.6%) were managed via stone extraction through the left hepatic duct stump, while 54 (68.4%) required common bile duct incision. Among them, 36 (45.6%) underwent T-tube drainage, and 16 (20.3%) underwent primary suture of the common bile duct. The bleeding score of liver surgical field is shown in Table 4.^[2]^ Three cases (3 %) of cholangiocarcinoma were accidentally found during operation, including 1 case in CLCVP group and 2 cases in NCLCVP group, all of which were located at the confluence of left hepatic duct. Preoperative imaging (CT/ MRI) showed irregular thickening of intrahepatic bile ducts, but no malignant diagnosis was confirmed. All 3 patients underwent extended left hepatectomy and regional lymph node dissection. The postoperative pathological diagnosis was intrahepatic cholangiocarcinoma.

**Table 3:** Comparison of intraoperative conditions between the 2 groups.

	CLCVP group (n = 45)	NCLCVP group (n = 55)	*P*-value
Conversion, n (%)	3 (7%)	4 (7%)	.906
Urine output (mL)	288 ± 78.15	266.91 ± 41.36	.107
Estimated blood loss (mL)	185.56 ± 71.21	260.00 ± 122.63	**<.001**
Operative time (min)	303.00 ± 40.15	328.18 ± 51.43	.**007**
Transfusion, n (%)	10 (22%)	15 (27%)	
Combined with CBD stones, n (%)			
LHDE	10 (22%)	15 (27%)	.562
LCBDE	25 (56%)	29 (53%)	.778
Post-LCBDDE, n (%)			
T-tube drainage	14 (31%)	22 (40%)	.357
Primary closure	9 (20%)	7 (13%)	.324
SBP during PTC (mm Hg)	99.00 ± 6.46	108.31 ± 8.06	**<.001**
MAP during PTC (mm Hg)	76.11 ± 4.01	72.22 ± 2.07	<**.001**
Central venous pressure (mm Hg)			
Anesthesia completed	8.42 ± 0.60	8.44 ± 0.58	.874
During PTC	4.42 ± 1.01	9.59 ± 0.74	<**.001**
After liver resection	8.11 ± 0.67	9.28 ± 0.78	<**.001**
Serum lactate level			
Anesthesia completed	1.07 ± 0.18	1.03 ± 0.16	.190
During PTC	1.57 ± 0.14	1.39 ± 0.10	<**.001**
After liver resection	1.81 ± 0.07	1.42 ± 0.07	<**.001**

CBD = common bile duct, CLCVP = controlled low central venous pressure, LCBDE = laparoscopic common bile duct exploration, LHDE = laparoscopic hepatic duct exploration, MAP = mean arterial pressure, NCLCVP = normal CVP, PTC = portal trail clamping, SBP = systolic blood pressure.

**Table 4:** Bleeding score of the hepatic surgical field.

Bleeding score	CLCVP group (n = 45)	NCLCVP group (n = 55)	*P*-value
0 = no bleeding (n)	5	2	.145
1 = minor bleeding, no aspiration required (n)	14	6	**.012**
2 = minor bleeding, aspiration required (n)	18	26	.466
3 = minor bleeding, frequent aspiration required (n)	4	10	.183
4 = moderate bleeding, visible only aspiration (n)	4	11	.122
5 = severe bleeding, frequent aspiration required (n)	0	0	

CLCVP = controlled low central venous pressure, NCLCVP = normal CVP.

Postoperative data are summarized in Table 5. Three patients (3 %) had postoperative drainage tube bleeding, and the bleeding was controlled after the use of hemostatic drugs. One patient (2 %) in the NCLCVP group had postoperative abdominal bleeding and bile leakage, and the patient eventually died of septic shock due to uncontrolled blood loss. One patient (1 %) had abdominal hemorrhage, and hepatic artery angiography showed hepatic artery hemorrhage, and was discharged after interventional embolization. Postoperative cholangitis occurred in 13 (13 %) patients and improved after anti-infective treatment. One patient (1 %) had residual stones in the liver after operation and was completely removed by the second operation. Postoperative bile leakage occurred in 10 patients (10 %), and all patients were discharged after extubation. Pleural effusion occurred in 35 (35 %) patients, and they were discharged smoothly after treatment. The postoperative hemoglobin level in the CLCVP group was higher than that in the NCLCVP group (106.87 ± 7.40 vs 99.27 ± 5.03 g/ L, *P* < .001). The levels of alanine aminotransferase (148.66 ± 22.11 vs 138.74 ± 20.67 U/ L, *P* = .024) and total bilirubin (56.12 ± 11.40 vs 51.19 ± 11.59 μmol/ L, *P* = .035) in the NCLCVP group were higher than those in the CLCVP group. The GFR in CLCVP group [98.75 ± 2.92 vs 97.77 ± 1.75 mL/ (min · 1.73 m^2^), *P* = .041] was higher than that in NCLCVP group. There were no significant intergroup differences in postoperative hospital stay, bile leakage, drainage tube removal time, wound infection, pneumonia, abdominal hemorrhage, reoperation, or in-hospital mortality. The remaining patients were discharged uneventfully postoperatively.

**Table 5:** Postoperative outcome data of 2 groups of patients.

	CLCVP group (n = 45)	NCLCVP group (n = 55)	*P* value
Postoperative hospital stay, d	10.96 ± 7.40	11.51 ± 1.89	.106
Time to removal of drain, d	5.80 ± 1.18	5.85 ± 1.35	.832
Intra-abdominal hemorrhage, n (%)	2 (4.4)	3 (5.5)	.818
Cholangitis, n (%)	5 (11.1)	8 (14.5)	**.611**
Reoperation, n (%)	0	1 (1.8)	.363
Bile leakage, n (%)	4 (8.9)	7 (12.7)	**.749**
Wound complication, n (%)	4 (8.9)	7 (12.7)	.542
Mortality, n (%)	0	1 (1.8)	.363
Residual stone, n (%)	0	1 (1.8)	.363
Stone recurrence, n (%)	2 (4.4)	4 (7.2)	**.453**
Pleural effusion, n (%)	15 (33.3)	20 (36.4)	.752
Hemoglobin, g/L	106.87 ± 7.40	99.27 ± 5.03	<**.001**
Prothrombin time, s	13.79 ± 1.76	13.34 ± 0.81	.119
Alanine transaminase, U/L	138.74 ± 20.67	148.66 ± 22.11	**.024**
Aspartate transaminase, U/L	153.89 ± 26.34	161.09 ± 18.34	.111
Total bilirubin, μmol/L	51.19 ± 11.59	56.12 ± 11.40	**.035**
Albumin, g/L	35.41 ± 2.56	35.43 ± 3.44	.982
Scr, μmol/L	75.38 ± 6.58	74.47 ± 8.38	.556
BUN, mmol/L	6.80 ± 1.34	7.13 ± 1.32	.216
GFR [mL/(min·1.73 m^2^)]	97.77 ± 1.75	98.75 ± 2.92	**.041**

BUN = blood urea nitrogen, CBD = common bile duct, CLCVP = controlled low central venous pressure, GFR = glomerular filtration rate, NCLCVP = normal CVP, PG = previous gastrectomy, Scr = serum creatinine.

Follow-up outcomes are presented in Table 5. With a median follow-up duration of 11.5 months (range, 5–72 months), no significant intergroup differences were observed in terms of stone recurrence or postoperative cholangitis. The follow-up completion rate was 100 %. All patients completed the outcome assessment through outpatient or telephone.

## 5. Discussion

Laparoscopic hepatectomy is an effective and widely used surgical method for the treatment of various benign and malignant liver diseases.^[16]^ However, the liver has a unique anatomical structure and a rich blood supply, making laparoscopic hepatectomy incredibly challenging. Excessive blood loss during laparoscopic hepatectomy is highly correlated with perioperative morbidity and mortality.^[17]^ In addition, intraoperative blood transfusion can significantly increase the incidence of complications after liver surgery.^[18]^ Therefore, it is necessary to find an effective and safe method to reduce bleeding during laparoscopic hepatectomy.

Previously, a prior history of abdominal surgery was regarded as a contraindication for laparoscopic procedures. Severe abdominal adhesions and obscured anatomical structures posed risks of intraoperative hemorrhage, bile duct injury, gastrointestinal trauma, and other abdominal organ injuries. Nevertheless, with advancements in laparoscopic techniques and accumulation of surgical experience, an increasing number of patients with a history of abdominal surgery are now being managed with laparoscopic approaches. Studies have confirmed that laparoscopic cholecystectomy, laparoscopic common bile duct exploration and laparoscopic hepatectomy are safe and feasible for patients with a history of abdominal surgery.^[19–21]^

Hepatolithiasis is one of the common benign lesions of the liver and has the characteristics of high recurrence. The intrahepatic bile ducts ramify within the liver parenchyma in an arborizing pattern, with well-defined demarcations between the biliary trees of individual liver segments. Intrahepatic stones and their surrounding lesions also exhibit distinct segmental distribution.^[10]^

Makuuchi first introduced the concept of anatomical hepatectomy,^[22]^ a surgical strategy aimed at addressing the high rates of postoperative stone residue and recurrence.^[23]^ The hepatic veins serve as critical anatomical landmarks for such resections.^[22]^ This study focused on left hepatectomy, which can be classified into 2 approaches: MHV-guided anatomical left hepatectomy and traditional non-MHV-guided anatomical left hepatectomy. In the former, MHV is identified during liver parenchyma transection, whereas the latter maintains a hepatic resection margin approximately 1 cm left of the MHV to preserve more liver tissue and avoid massive hemorrhage from potential MHV injury. Several institutions have reported comparisons between MHV-guided and non-MHV-guided anatomical hemihepatectomy, demonstrating the safety and feasibility of the MHV-guided approach.^[8–10]^

Previously, the main method of controlling intraoperative bleeding during laparoscopic hepatectomy was Pringle maneuver. Although Pringle maneuver is considered to be a method to reduce bleeding during hepatectomy, hepatic vein bleeding is inevitable.^[3]^ At the same time, the obstruction and opening of the hepatic portal vein can lead to liver ischemia-reperfusion injury.^[24]^ Severe hypotension and long-term obstruction of hepatic blood flow can lead to severe hypoxia and ischemia of the liver,^[25]^ which ultimately affects the recovery of liver function and even leads to postoperative liver failure. In addition, by optimizing the preoperative nutritional status of patients, reducing intraoperative blood loss and blood transfusion rate, the incidence of postoperative liver failure can be reduced.^[17,26,27]^ Therefore, anesthesiologists should pay more attention to the protection of liver function and reduce the damage of organ hypoperfusion and hypoxia to the liver.

Previous studies have reported that controlling low CVP < 5 cmH_2_O or 3 cmH_2_O can significantly reduce hepatic vein bleeding.^[5,28]^ Yu et al^[2]^ thought that the lower CVP during hepatectomy could provide the best surgical field of vision, and had no significant effect on the amount of intraoperative blood loss. A global survey has shown that 85% of centers use a strategy of limiting intraoperative infusion volume during hepatectomy to reduce CVP and reduce intraoperative blood loss.^[29]^ It can be seen from the surgical field bleeding score that the surgical field bleeding score of the CLCVP group was 1 point (slight bleeding, no suction), which was significantly higher than that of the NCLCVP group.

The main measures of CLCVP include limiting fluid intake and dilating arterial blood vessels with vasoactive drugs, so as to reduce effective circulating blood volume and return heart blood volume.^[2]^ This leads to a decrease in cardiac output and insufficient organ perfusion. If this state lasts for a long time, tissue and organ damage may occur and affect postoperative recovery. Therefore, in the process of laparoscopic hepatectomy, CVP should be controlled at a low level by limiting fluid intake and other measures. At the same time, it is also necessary to avoid MAP reduction and ensure the perfusion of important organs.

Regarding the optimal control value of CVP, studies have shown that controlling CVP < 1 cmH_2_O during lobectomy and infusion of norepinephrine to maintain arterial pressure can increase the level of lactic acid in the body, suggesting that insufficient organ perfusion may be related to CVP < 1 cmH_2_O g.^[30]^ Controlling CVP at 2.1 to 3 cmH_2_O can reduce bleeding and prevent damage to important organs. This level has no significant effect on the body’s oxygen supply, oxygen consumption and oxygen metabolism rate.^[31]^ When CVP is lower than 2 cmH_2_O, the oxygen supply, oxygen consumption and oxygen metabolic rate are significantly reduced.^[32]^ Similarly, in this study, the CVP of patients in the CLCVP group was not continuously reduced, but was controlled at 2 to 5 cmH_2_O, which not only reduced bleeding, but also prevented damage caused by insufficient perfusion of important organs to a certain extent. The postoperative GFR in the CLCVP group was higher than that in the NCLCVP group, which was considered to be caused by the increase of intraoperative lactic acid. However, the GFR of all patients returned to normal before discharge.

After the first hepatic portal vein was blocked during the operation, the bleeding mostly originated from the hepatic vein and hepatic sinus. As the pressure of the inferior vena cava decreased, the pressure of the hepatic vein and hepatic sinus also decreased. Studies have shown that the pressure of the inferior vena cava is significantly consistent with the pressure of the right atrium. The decrease of CVP can reduce the pressure of hepatic vein and hepatic sinus.^[33]^ Therefore, controlling CVP at a low level during laparoscopic MHV-guided anatomical resection of the left liver is conducive to blood returning to the hepatic vein system, thereby reducing intraoperative bleeding and reducing the difficulty of surgery.^[34]^ Similar results were obtained in this study, that is, the intraoperative blood loss in the CLCVP group was reduced, which made the surgeon’s surgical field clearer, the operation time shorter, and the hepatic portal vein obstruction time shorter, thereby reducing the overall operation time and anesthesia time.

Venous gas embolism is an important complication of laparoscopic hepatectomy. When the hepatic vein fissure is large, the gas in the abdominal cavity is easy to enter the systemic circulation through the hepatic vein, resulting in gas embolism.^[35]^ Studies have found that in liver surgery, the incidence of gas embolism is significantly increased when the artificial pneumoperitoneum pressure/ CVP is increased. It is suggested that in the operation around the larger hepatic vein, the pneumoperitoneum pressure or CVP should be appropriately reduced to reduce the pneumoperitoneum pressure/ CVP ratio and avoid severe gas embolism.^[36]^ On the other hand, although CLCVP technology can reduce bleeding, shorten the operation time, hepatic portal vein occlusion time and hospitalization time, promote the recovery of patients and improve the postoperative survival rate, there is also a risk of insufficient perfusion of important organs.^[35,37–41]^

Abdominal adhesion remains a significant surgical challenge. Based on our experience, establishing pneumoperitoneum is critical to minimize abdominal trauma. The Veress needle should be inserted at a minimum of 3 cm from the original incision. If pneumoperitoneum cannot be successfully established, the Hasson technique can be employed,^[42]^ which helps avoid intestinal and vascular injuries. When separating abdominal adhesions, patience and caution are essential, following the principle of proceeding from easy to difficult areas. In cases of dense adhesions, dissection should be performed close to the liver surface to prevent gastrointestinal damage.

MHV-guided anatomical left hemihepatectomy initially exposes MHV and cuts the liver parenchyma along it, which can reduce unnecessary damage during surgery. The transverse surface of the liver coincides with the parahepatic venous space, which is a relatively non-vascular interface without Glisson branch, which can avoid the injury and bleeding of the Glisson system during liver transection. This is one of the reasons for less intraoperative bleeding and low incidence of postoperative bile leakage in the MHV group.

Anatomy and exposure of MHV is also one of the keys to surgery. Our experience is to use ultrasound to accurately locate the MHV trunk during the operation, mark the liver surface of the MHV trunk with an electric hook, and then use an ultrasonic scalpel to cut the liver parenchyma 2 to 3 cm away from the anterior and lower edges of the liver along the marking line to find the branches around the MHV. Starting from this position, the MHV trunk and its branches were isolated and exposed until the second porta hepatis. If intraoperative MHV bleeding occurs, the degree of bleeding should be evaluated: for MHV small sieve aperture bleeding, gauze, hemostatic gauze, gelatin sponge, bipolar electrocoagulation, etc, can be used to stop bleeding; for slightly larger MHV trunk rupture bleeding, use 5-0 Prolene suture to stop bleeding. In this study, the operation time of the CLCVP group was lower than that of the NCLCVP group. We believe that the former has less bleeding and less hemostasis time, thus shortening the operation time.

Conversion to laparotomy when the following situations occur: severe abdominal adhesions lead to unclear anatomical structure; hepatic vein tear bleeding, unable to control; intraoperative hypotension (MAP 15 minutes; air embolism).

This study validated the safety of CLCVP in a specific population with a history of intrahepatic bile duct stones and upper abdominal surgery, aligning with Western findings. Notably, Asian patients exhibit a higher prevalence of intrahepatic bile duct stones, necessitating more extensive anatomical resection.^[7,43]^ This study enrolled patients with intrahepatic bile duct stones, Child-Pugh class A or B, who had a history of upper abdominal surgery. CLCVP should be used with caution in patients with poor liver function. CLCVP can also be attempted for patients with benign and malignant tumors confined to the left and right lobes of the liver.

After 6 years of research, we found that the rate of conversion to laparotomy was higher in the first 3 years (5/ 100 vs 2/ 100, *P *= .08), suggesting that the surgeon’s experience and understanding of anatomy were crucial to the success of laparoscopic hepatectomy. With the progress of the study, the amount of bleeding during the operation was gradually controlled.

One-year follow-up after operation showed that the residual liver volume of the 2 groups was higher than that before operation, and the Child-Pugh grade was maintained at grade A. Serum ALT and AST levels returned to normal range during postoperative follow-up, suggesting that CLCVP technique had no significant adverse effects on long-term liver parenchymal function. Future research could include a detailed evaluation of patients’ postoperative quality of life using the SF-36 scale.

Our study has some limitations: 1. The sample size of CLCVP patients is relatively small (45 cases in the CLCVP group), and a multicenter study and a larger sample size are needed to verify our findings; 2. Since this study is a retrospective study, the results may have selection bias and need to be further confirmed by multicenter clinical trials; the follow-up time of long-term stone recurrence rate data was short (median 11.5 months), and prospective studies will be carried out in the future for further evaluation.

## 6. Conclusions

Overall, the safety, efficacy, and feasibility of the CLCVP approach in MHV-guided laparoscopic anatomical left hemihepatectomy were validated for patients with left intrahepatic bile duct stones and a prior history of upper abdominal surgery. It not only shortens the operation time, but also reduces intraoperative bleeding, thereby reducing the incidence of perioperative complications and mortality.

## Acknowledgments

We would like to thank the contributions of all of the patients in our retrospective study.

## Author contributions

**Conceptualization:** Lu Fang, Jian Huang.

**Data curation:** Huizhen Chen, Weihua Pan.

**Formal analysis:** Dingxiu He, Jinghang Liu, Huizhen Chen.

**Investigation:** Yuting Fan, Jinghang Liu.

**Project administration:** Yuting Fan.

**Resources:** Weihua Pan.

**Software:** Jian Huang.

**Supervision:** Wei Hu.

**Visualization:** Jian Huang.

**Writing – original draft:** Wei Hu, Longjian Ran, Lu Fang, Jian Huang.

**Writing – review & editing:** Lu Fang.
